# Guided Evolution of Recombinant *Bombyx mori* Acetylcholinesterase II by Homology Modeling to Change Pesticide Sensitivity

**DOI:** 10.3390/ijms19113366

**Published:** 2018-10-27

**Authors:** Jun Cai, Bingfeng Wang, Jiadong Li, Zijian Chen, Meifang Rao, Serge Muyldermans, Xiude Hua, Xi Xie, Hong Wang, Jinyi Yang, Zhenlin Xu, Yudong Shen, Yuanming Sun

**Affiliations:** 1 College of Food Science, South China Agricultural University, Guangzhou 510642, China; corrinej@stu.scau.edu.cn (J.C.); ljdong135@163.com (J.L.); czj1q2w3e4r5t@stu.scau.edu.cn (Z.C.); rmfrwl@163.com (M.R.); xix004@mail.usask.ca (X.X.); yjy361@163.com (J.Y.); jallent@163.com (Z.X.); shenyudong@scau.edu.cn (Y.S.); ymsun@scau.edu.cn (Y.S.); 2 Guangdong Province Key Laboratory of Food Quality and Safety, South China Agricultural University, Guangzhou 510642, China; wbfeng@scau.edu.cn; 3 College of Materials and Energy, South China Agricultural University, Guangzhou 510642, China; 4 Laboratory of Cellular and Molecular Immunology, Vrije Universiteit Brussel, Pleinaan 2, 1050 Brussels, Belgium; serge.muyldermans@vub.be; 5 College of Plant Protection, Nanjing Agricultural University, Nanjing 210095, China; huaxiude@njau.edu.cn; 6 Department of Food and Bioproduct Sciences, University of Saskatchewan, Saskatoon, SK S7N 5A8, Canada

**Keywords:** docking, bottleneck, molecular dynamic simulation, side chain

## Abstract

Acetylcholinesterase (AChE) has been widely used for the detection of organophosphate and carbamate pesticides, due to its high sensitivity and low limit of detection to the presence of pesticides. The homology modeled recombinant *Bombyx mori* Acetylcholinesterase II (r*Bm*-AChE II) and docking results with multiple pesticides inferred that Y398, located at the bottleneck of the active site gorge, might be important for enzyme sensitivity. Thus, three mutants (Y398G, Y398F, Y398W) were constructed and exhibited different enzyme activities and sensitivities. The results showed that Y398W possessed a remarkably increased enzyme activity, while Y398F had a significant reduction. The Y398F has an approximately 2-fold lower IC50 for some pesticides than the wild type enzyme, indicating a higher sensitivity. With the detailed investigation of the conformations of computer simulation, we propose that for the positively charged and small substrate ATChI, a larger side chain at position 398 improves the fixation of the substrate in an appropriate conformation for catalysis. For bulky substrates such as pesticides, the diffusion in the active site gorge may be related to the enlargement of the bottleneck by having proper orientations more easily. In addition, a more hydrophobic side chain at the bottleneck seemed to be beneficial for ligand diffusion.

## 1. Introduction

Acetylcholinesterase (AChE) is a serine hydrolase that terminates the transmission of nerve pulses in the nervous system by rapidly catalyzing the hydrolysis of acetylcholine [1]. It is well established that organophosphate (OPs) and carbamate (CBs) pesticides result in the irreversible phosphorylation and carbamylation of the serine at the active site, provoking serious toxicity to organisms [2]. Nevertheless, this characteristic provided an elegant method to detect OPs and CBs via an enzymatic analysis. The holding hypothesis is that OPs and CBs hinder the catalysis of substrates by AChE, thereby reducing the color change induced by the decomposition of the chromogenic reagent. Enzymatic analysis based on AChE is a rapid semi-qualitative method, which is extensively used in on-site tests of OPs and CBs contaminating agricultural products. As early as 1976, Konrad et al. [3] had used this labor-saving and rapid method to detect OPs residues in milk. Nowadays, various modified enzymatic analysis methods have been developed, including biosensors based on gold nanoparticles and sol-gel enzymes [4,5]. The AChE, being the critical reagent of the enzymatic analysis can be obtained from different sources. A diverse sensitivity to pesticides originating from the difference of substrate affinities and rates of phosphorylation was noted from the comparative study of AChE from various species [6]. The tests revealed that insect AChE is more susceptible to OPs and CBs than the AChE from other species [7]. Among the insects, the AChE of silkworm, *Bombyx mori*, which was fed artificially without pesticides for many years, shows advantage in sensitivity [8].

Since AChE from different sources was successfully cloned and expressed recombinantly [9,10], it became possible to search for improved variants obtained after genetic engineering methods such as site directed mutagenesis and molecular evolution techniques. Over the years, the AChE studies aimed at understanding the resistance towards pesticides, the interactions with drugs or inhibitors, and the development with superior sensitivity to OPs and CBs. For ligand binding in the AChE active center, the substrates or inhibitors have to cross the deep gorge, guided by a series of noncovalent interactions, followed by the chemical reaction with the catalytic triad at the bottom of the gorge [11]. Since the catalytic triad and its action mechanism are highly conserved, the evolution of AChE has always been focusing on the gorge and the path followed by the ligand involving analysis of noncovalent interactions such as π–π interactions, π–cation interaction, hydrogen bonds, binding force, etc. The AChE inhibitor or drug design, molecular analysis including dynamic simulation, docking and energy calculation were successfully and widely used, revealing the utility of *in silico* structural analysis. Antosiewicz et al. [12] analyzed the binding energy and interactions of *Torpedo californica* AChE with particular drug molecules and concluded that galanthamine, which binds to Phe330, Trp84, and Asp72, shows higher binding affinity than other molecules devoid of such interactions. Ambure et al. [13] reported a detailed screening by molecular docking of potential inhibitors of the human AChE. As a result of a bioinformatic analysis, proposals for the evolution of the AChE sensitivity to pesticides could be made. As such, Boublik et al. [14] substituted the residues as suggested by a docking study of insecticides to the *Drosophila* AChE active site (with known 3D-structure) and obtained the L370F mutant that was 14-fold more sensitive to Carbaryl and three other insecticides than the wild AChE. However, these investigations are dependent on the availability of the crystal structures of AChE to provide accurate structural information. For proteins where the crystal structure is not available, the amino acid sequence information or the predicted 3D-structure obtained by molecular modeling could be used as starting point for a rational design of variants. Indeed, an A303S/G329A/L554S mutant of *B. mori* AChE with a similar enzyme activity but lower pesticide sensitivity than wild type AChE was created based on sequence information only, by Ju-Mei et al. [15]. Tan et al. [16] generated Y327D and I374D mutants of *Musca domestica* AChE after sequence alignment and 3D structure modeling, which lead to a 14–16-fold improvement of the sensitivity to particular pesticides. Devi et al. [17] performed molecular modeling on *Blattella germanica* and docking with pesticides, to conclude that monochrotophos, an organophosphorous insecticide and acaricide, would be potential drugs. Finally, Mutunga et al. [18] created novel carbamates after a structural modeling exercise on *Anopheles gambiae* AChE, which provoked greater enzyme sensitivity. Hence, the homology modeling method was regularly employed to identify the interaction mechanism of protein and ligands, or to predict the key amino acids for protein function. However, few research attempts employed guided evolution based by homology modeling on AChE, despite several examples exist for other proteins. Tholander et al. [19] demonstrated the importance of Glu316 and Arg627 in mammalian leukotriene A4, following their modeled structure analysis, mutagenesis and binding studies. The enzyme variants with mutations at those positions exhibited a 24- and 63-times increase in enzyme activity. In previous studies, the functions of residues in the active site gorge of AChE were extensively explored through site-directed mutagenesis and molecular dynamic simulation methods. These efforts provided firm basis of our project design and data analysis. It was demonstrated that the size and shape of the active center, and also the electrostatic characteristics always had significant effects on diffusion and binding of ligands [20,21]. Several investigations focused on the side chain function of F330 (numbered in *Torpedo californica*), located at the bottleneck of the gorge and also acting as the component of the choline-binding site. It was suggested that increasing of the volume of the active site gorge by an F353A mutation in human AChE would cause an increased sensitivity for some inhibitors, while a decreased inhibition rate was observed with carbamate inhibitors due to critical effects of π–π interactions [22]. Katalinić et al. speculated that the replacement of the aromatic side chain (Y337A) would produce great interference with oxime’s pyridinium ring stabilization in mouse AChE [23]. Moreover, the absence of the F330 side chain might affect inhibitions by changing the conformation of other residues [24] or by eluting the inhibitor [25]. Based on these former experiments, the steric hindrance should be in evolutionary design of AChE.

In this report, homology modeling and docking were applied to guide the challenging sensitivity improvement of recombinant *Bombyx mori* type II AChE (r*Bm*AChE II). In a previous study, we cloned and codon optimized the *Bombyx mori* type II AChE, which was verified to be more sensitive to the commercial fish head AChE (in preparation).

We predicted a 3D-structure of r*Bm*AChE II and carried out the molecular docking of r*Bm*AChE II with substrates acetylthiocholine iodide (ATChI) and other pesticides. Y398 (F330 in *Torpedo californica*), which is located at the bottleneck of the active site, was predicted to be critical. On the basis of previous studies, the amino acid at this position was substituted independently by three residues (Y398G, Y398F, Y398W), to alter its size, the aromatic side chain, and the hydrophobicity. Furthermore, since the mutation caused changes in both enzyme activity and sensitivity to pesticides, steered molecular dynamic simulation was conducted to clarify the pesticide binding mechanism.

## 2. Results

### 2.1. Simulation of 3D-Structure and Docking

**Figure 1:**
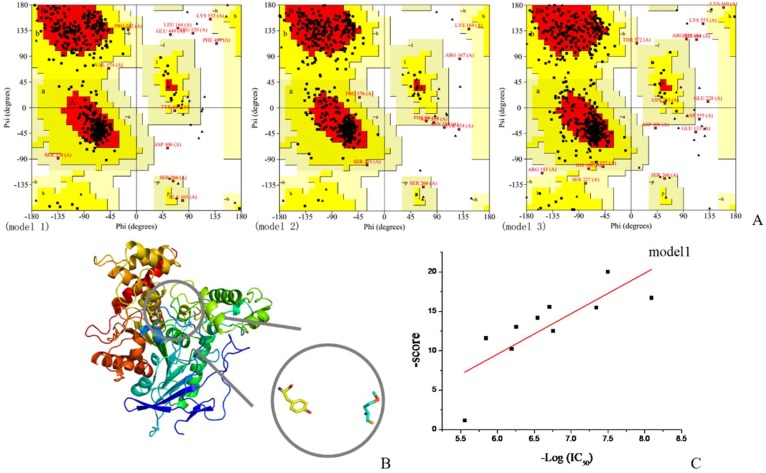
Fitted curve of the most appropriate models. (**A**) Ramachandran plots of three models, (**B**) model 1 which was chosen for further studies, and (**C**) scatter plot and standard curve based on docking scores and IC_50_.

The Swiss-model online server was used to obtain a 3D structure of r*Bm*AChE II. Three structural models were returned and the Ramachandran plots were drawn by PROCHECK (Figure 1A). For model 1, 86.1% of the residues were laying in the most favored regions, which turned out to be the highest score among the three models. For the disallowed regions, model 1 showed 1.3% of its residues in this region, while model 2 has 0.6%. The results indicate that models 1 and 2 are of a higher quality than model 3, which has only 72.7% residues in the most favored regions and 1.7% residues in disallowed regions. Based on the fitted curves of docking scores and IC_50_, the most suitable model with *R*^2^ = 0.596 was chosen (Figure 1B,C). The root mean square deviations (RMSD) between the chosen model and *D. Melanogaster* AChE 3D structure (PDB ID: 1QO9) was 0.586 reflecting a very high congruence to the template structure.

**Figure 2:**
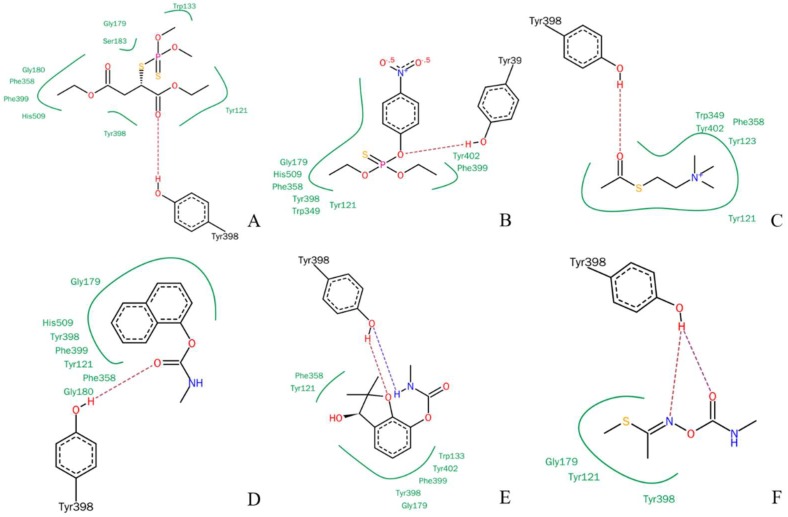
Planar schematic representation of the LeadIT solutions for pesticides in the active site pocket of r*Bm*AChE II. (**A**) Malathion, (**B**) parathion, (**C**) ATChI, (**D**) carbaryl, (**E**) Carbofuran-3-hydroxy, and (**F**) methomyl.

The LeadIT program was used to simulate the interactions between r*Bm*AChE II and various substrates. The planar schematics revealed that all substrates formed hydrogen bonds with Y398, which is located at the edge of the choline binding site (Figure 2). We also know from our analysis by the on-line server POCASA 1.1 that the active site pocket of the mutants (Figure 11B) differs widely, depending on the side chain of residue 398 (Y/G/F/W). As calculated by the server, the volumes of the pockets are 104 Å^3^ (Y398), 173 Å^3^ (Y398G), 98 Å^3^ (Y398F), and 67 Å^3^ (Y398W).

### 2.2. Plasmid Construction and Transformation

Plasmids with gene *Bm*ace2 mutated at position Y398 into G, W, and F were successfully constructed, confirmed by sequencing and electrotransformed into GS115 competent cells, along with the wild type plasmid. Electrotransformation of wild type and mutant pPIC9K-*Bm*ace2 generated dozens of clones. Expression of the clones in 96-deep well plates indicated the presence of several enzymatically active clones, which were chosen for further investigation.

### 2.3. Determination of the Gene Copy Number of Bmace2 by q-PCR

To avoid effects of differences in *Bm*ace2 copy numbers that may lead to dissimilarities in enzyme yield and activity, we performed a q-PCR to identify clones with comparable number of gene copies. Standard curves with two pairs of primers showed that the correlation coefficients were greater than 0.98 and amplification efficiencies were between 0.9–1.2. Melting curves of the amplicons obtained with each pair of primers displayed a single peak. These results indicated that primers of GAPDH and *Bm*ace were specific and appropriate for q-PCR. Clones exhibiting *Bm*ace2 enzyme activities were chosen as template for q-PCR. Based on the 2^−ΔΔ*C*t^ method, we calculated the relative copy number of the mutated *Bm*ace gene within the different clones (Table 1). The clones Y-33, G-15, F-16, and W-10 displayed a similar relative copy number, closely equal to 1.

### 2.4. Analysis of Enzyme Activity and Inhibition by Physostigmine

**Figure 3:**
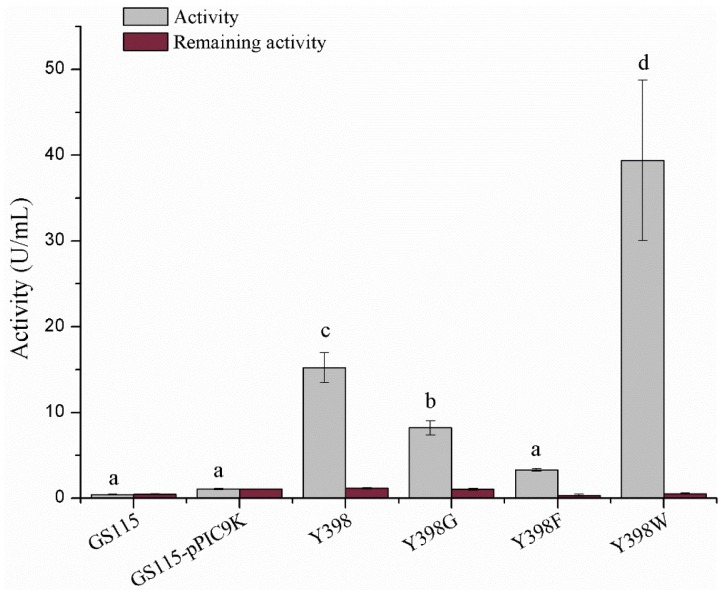
Enzymatic activity and inhibition by physostigmine. GS115 and GS115-pPIC9K refer to the supernatant of *Pichia p.* GS115 without pPIC9K plasmid and *Pichia p.* GS115 with pPIC9K but no r*Bm*AChE II gene. The lowercase letters correspond to the significance (*t*-test, *p* < 0.05). The enzymatic activities various of labelled “a” show no significant difference; the enzymatic activities marked “b, “c” and “d” show significant difference to all others.

To assess the distinctive enzymatic activity of the r*Bm*AChE II mutants and their sensitivity to pesticides, we expressed the enzymes (wild type and the three mutants). Since the copy number of the *Bm*ace gene is identical in all our clones and since the growth of *Pichia* was similar for all clones, we expect that the supernatant of lysed cells will contain a comparable amount of enzyme. Therefore, the difference in enzyme activity will be largely dictated by the actual mutation. Wild type Y398 showed an enzyme activity of 15.23 ± 1.75 U/mL, whereas Y398G and Y398F displayed a significantly lower enzymatic activity of 8.22 ± 0.82 and 3.31 ± 15 U/mL. Remarkably, Y398W has an increased activity of 38.26 ± 5.73 U/mL (Figure 3). All four enzyme variants could be inhibited by physostigmine.

### 2.5. Analysis of Pesticide Sensitivity

**Figure 4:**
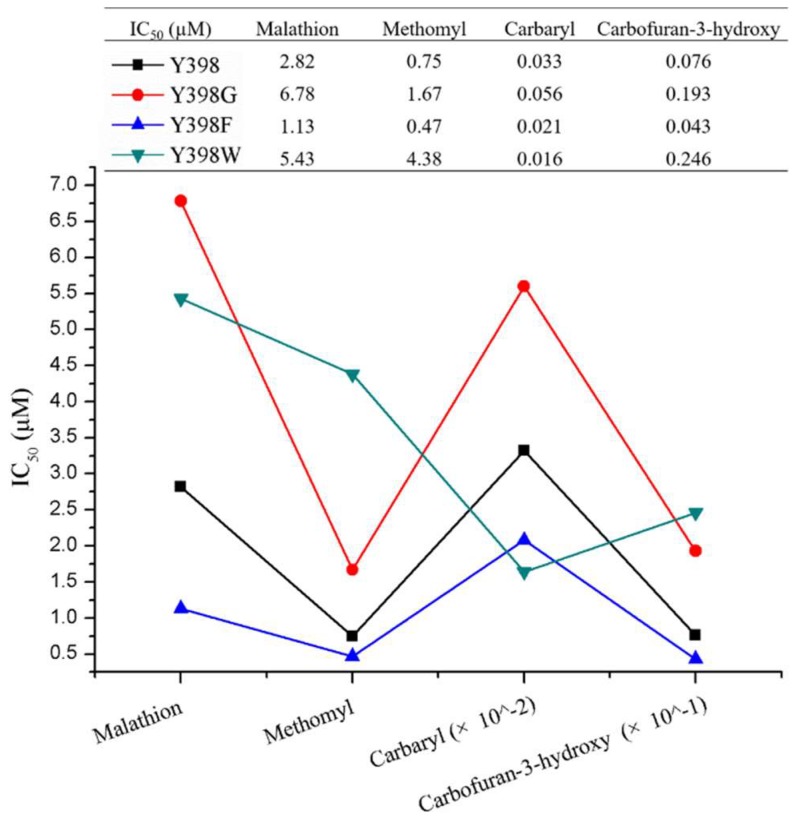
Comparison of inhibition of r*Bm*AChE II and its mutants by various pesticides.

The inhibition of r*Bm*AChE II mutants by various pesticides (malathion, methomyl, carbaryl, and carbofuran-3-hydroxy) was measured to assess the effect of the mutation on the enzyme sensitivity (Figure 4). Apparently, the Y398G and Y398W mutations had a 2- to 5-fold higher IC_50_ than Y398 (except Y398W to Carbaryl), while Y398F showed approximately a 2-fold lower IC_50_, which corresponds to a higher sensitivity to the pesticides under investigation.

### 2.6. Simulation of Molecular Dynamics

**Figure 5:**
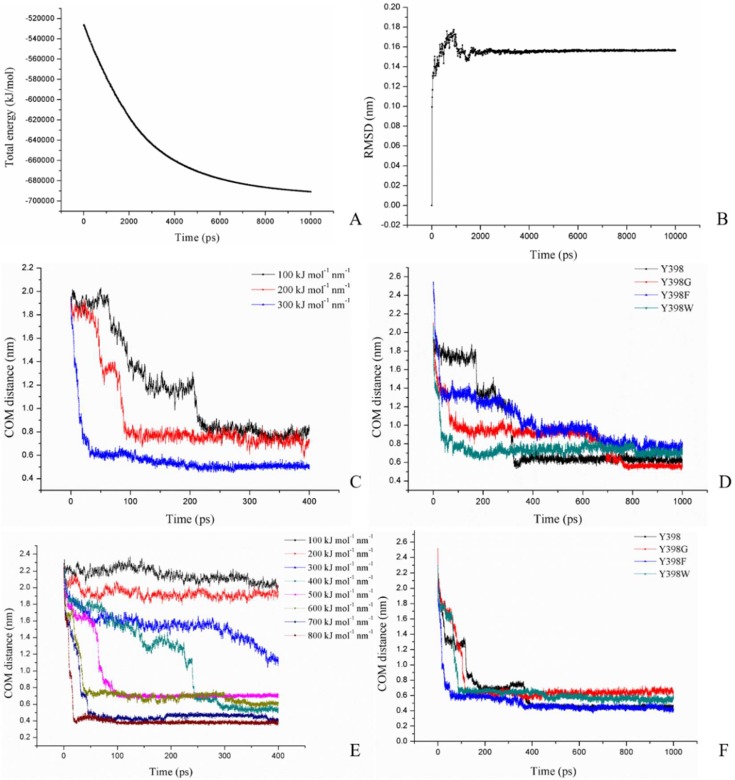
Molecular dynamic and pulling force results. (**A**) protein energy of model 1, (**B**) rms deviations of model 1, (**C**) center of mass (COM) distance changes of ATChI and S266 of model 1 under different constant pulling forces as indicated, (**D**) COM distance changes of ATChI and S266 with pulling force of 200 KJ·mol^−1^·nm^−1^ for the different mutants, (**E**) COM distance of Carbofuran-3-hydroxy and S266 under different constant pulling force, and (**F**) COM distance of Carbofuran-3-hydroxy and S266 with pulling force of 400 KJ·mol^−1^·nm^−1^ for the different mutants as indicated.

The four r*Bm*AChE II protein models (Y398, Y398G, Y398F, Y398W) were equilibrated under the amber99SB force field after EM using 500 ps PR, 500 ps NVT, 500 ps NPT, and 10 ns MD settings. The time dependent total energy and the root mean square deviations (RMSD) of Y398 are shown as a representative in Figure 5A,B, respectively. The total energy of model 1 decreased by 1.8 × 10^5^ KJ/mol and converged. The RMSD fluctuated during the first 2000 ps and stabilized around 0.155 nm.

To analyze the change in enzyme activity caused by the mutations, substrate ATChI was pulled from the entrance of the pocket towards the catalytic residue S266 at the bottom of the pocket. The ATChI and the active site S266 were set as the two pulling groups. Three constant pulling forces of 100, 200, and 300 KJ·mol^−1^·nm^−1^ were applied while the center of mass (COM) between the two pulling groups was recorded over time (Figure 5C). An appropriate constant force was supposed to be able to pull the substrate to the bottom of the active site, and at the same time it would show the force condition in the pulling process. In this case, we chose 200 KJ·mol^−1^·nm^−1^ as the appropriate constant force and conducted the same pulling force for 1 ns to the models of the different mutants (Figure 5D). For the Y398 model, the ATChI encountered two barriers, one at a distance of around 1.7 nm and one at 1.3 nm before meeting the S266 (at 0.6 nM) after 322.5 ps. The Y398F model exhibited a hindrance at 1.3 nm and 0.9 nm. A greater resistance occurred at these distances, which resulted in a longer residence time of ATChI in the active site gorge before reaching S266. This may result in a reduced enzyme activity of Y398F as observed experimentally. In contrast, for the Y398G model we noticed that the ATChI was confronted with a large barrier at 0.95 nm (whereby the ATChI would not be close enough to interact with S266) before it reaches the S266 with a COM of 0.5 nm. Such profile is likely to result in a slightly reduced activity of Y398G compared to wild type r*Bm*AChE II. Finally, applying the pulling force on the Y398W mutant led to a rapid migration of the ATChI through the bottleneck of the gorge. This substrate migration profile is expected to cause a significant increase in enzyme activity.

**Figure 6:**
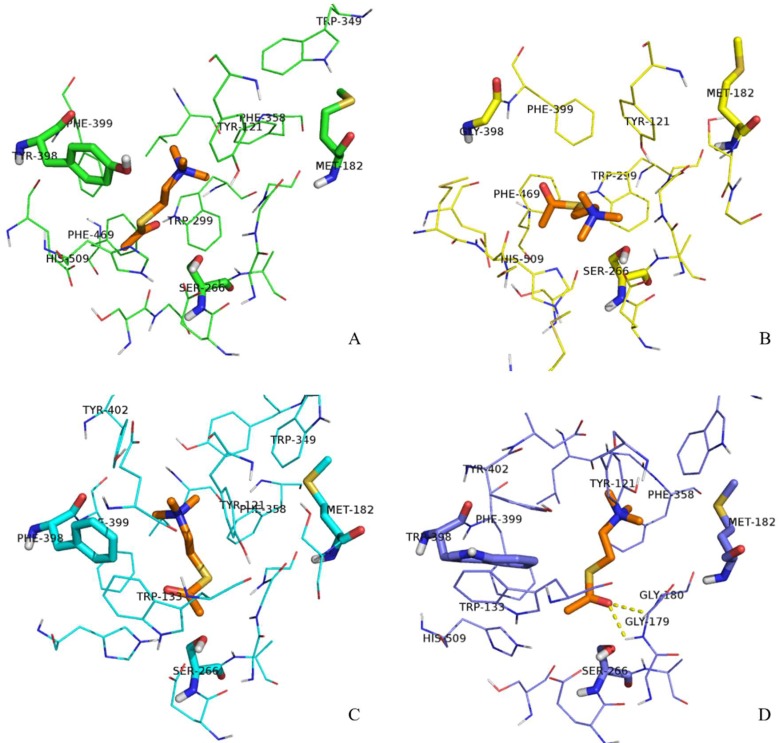
Conformations of ATChI after pulling under 200 KJ·mol^−1^·nm^−1^ for 1 ns. Substrate ATChI was presented by orange sticks. The structure of the gorge residues and catalytic S266 of wild type enzyme Y398 (**A**), Y398G mutant (**B**), Y398F mutant (**C**), and Y398W (**D**) mutant of r*Bm*AChE II are shown.

**Figure 7:**
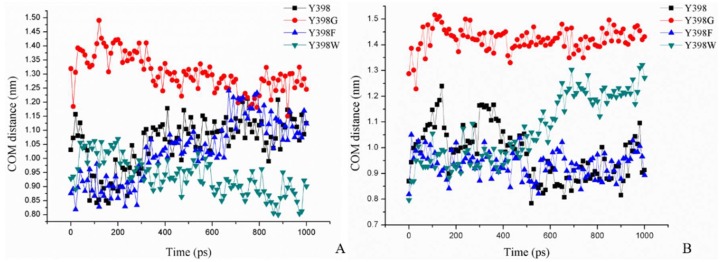
Minimum COM distance between Y/G/F/W398 and M182, (**A**) after the ATChI pulling process, and (**B**) after the Carbofuran-3-hydroxy pulling process.

Next, we decided to analyze the structure of the gorge around residue 398 during the substrate transit in more detail. The aromatic guidance of the substrate, especially by residues F399, Y402, H509, Y121, W133, and W349, is playing a primordial role in the process of ATChI transit. These residues are forming π–cation interactions with ATChI, altering its orientation by appropriate rotation of their side chains and eventually providing a position of the substrate that conforms its catalysis by S266. Figure 6 shows the conformation of the gorge residues of the wild type enzyme and its three mutants and ATChI after 1 ns pulling under 200 KJ·mol^−1^·nm^−1^. In the Y398 wild type enzyme model (Figure 6A), the quaternary nitrogen of ATChI seems to form the π-cation interaction with the Y398 side chain (aromatic ring and hydroxy). This interaction is accompanied with the “opening” of the bottleneck of the gorge (Figure 7A), so that ATChI could approach the catalytic S266 residue in suitable orientation for hydrolysis. In the model for Y398G (Figure 6B), the absence of side chain poses less steric hindrance at the bottleneck for the substrate, thereby providing a larger degree of freedom of ATChI at a cost of a less favorable guidance of this substrate in an optimal conformation for cleavage by the active site residue. This explains the lower activity of the Y398G mutant of r*Bm*AChE II enzyme to catalyze ATChI. The structural model of the Y398F mutant, which lacks only the hydroxy group, has its aromatic side chain (Figure 6C) in an analogical orientation to that of wild type Y398 (Figure 6A), although the enzymatic assay exhibited a significantly reduced activity. However, the conformations of the Y121 side chain after pulling the substrate for 500 ps and 1 ns is positioned at a widely different angle from that of the wild type enzyme. The Y121 is belonging to the peripheral anionic binding site (PAS) of the enzyme, and involved in a π–cation interaction with ATChI. The altered orientation of Y121 within the Y398F mutant will lead to steric hindrance, resulting in a less favorable positioning of the ATChI substrate to S266. Finally, in the Y398W model (Figure 6D), the G179 and G180, which are composing the “oxyanion hole”, are engaged in hydrogen bonding with the carbonyl oxygen of ATChI after a pulling force during 1 ns. Furthermore, the architecture of the Y398W, with obviously strong hydrophobicity, indicates a reduced flexibility with W133, F399, Y402 residues. As a result, electrostatic interactions between ATChI and both, Y121 hydroxy group and the F358 aromatic ring could be installed. These imposed restrictions on the ATChI flexibility makes its catalysis more efficient.

In Figure 7A, we show the minimum COM distance between Y/G/F/W398 and M182 (amino acid on opposite side of the gorge relative to amino acid at position 398) in the pulling process of ATChI. Clearly, while the gorge sizes of Y398 and the 398F mutant are very similar, that of Y398G is much wider and that of Y398W is narrower.

**Figure 8:**
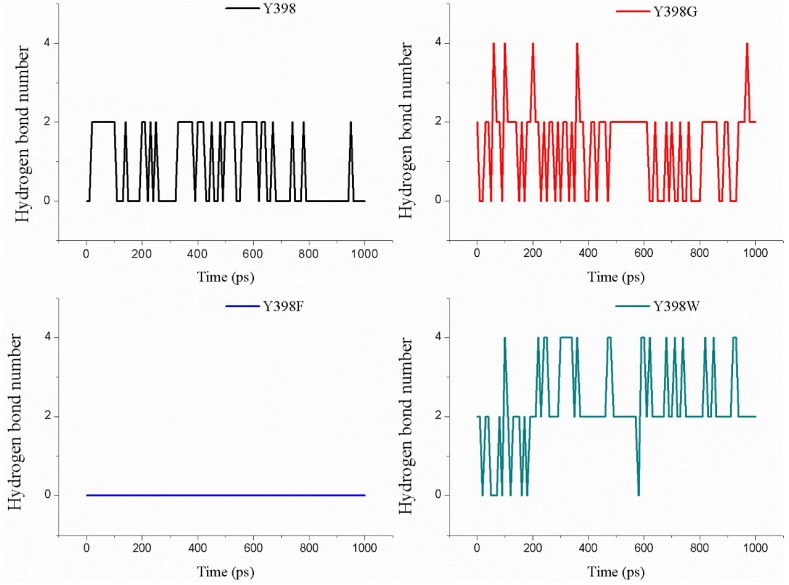
The amount of hydrogen bonds present between ATChI and the ezyme or its mutants during the pulling of ATChI for 1 ns.

The analysis of the occurrence of hydrogen bonds formed between the enzyme and the ATChI substrate is shown in Figure 8. The wild type enzyme seems to form between 0 and 2 H-bonds during the pulling of the substrate towards the catalytic S266. In contrast, this type of analysis indicates that Y398F fails to generate a H-bond at all times (Figure 8). The number of hydrogen bonds between substrate and enzyme Y298G and Y298W is higher than for the wild type enzyme. These results are also in line with the experimental enzymatic assays.

**Figure 9:**
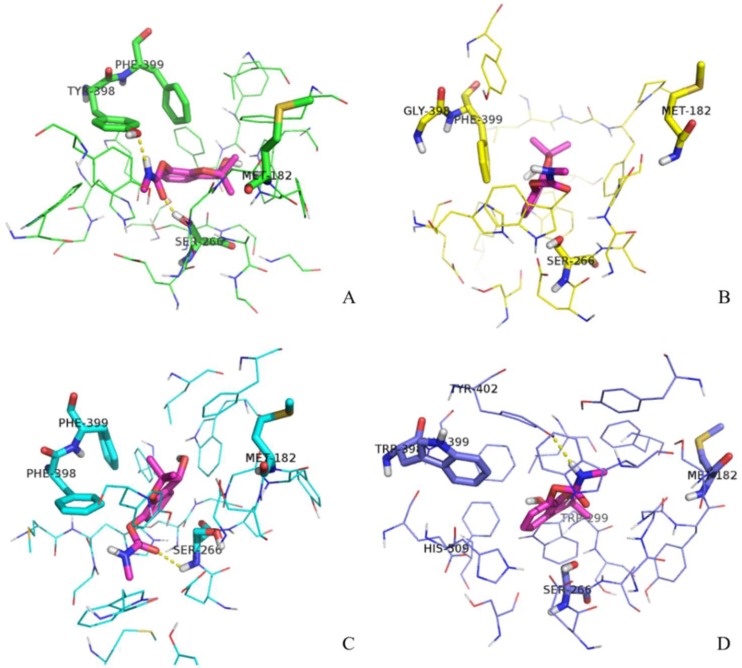
Conformations of Carbofuran-3-hydroxy after pulling for 1 ns at 400 KJ·mol^−1^·nm^−1^. Substrate Carbofuran-3-hydroxy is in pink stick representation. The structure of the gorge residues and catalytic S266 of wild type enzyme Y398 (**A**), Y398G mutant (**B**), Y398F mutant, (**C**) and Y398W mutant (**D**) of r*Bm*AChE II are shown.

We also applied various analogical constant-force pulling measurements to Carbofuran-3-hydroxy. After testing the pulling of this r*Bm*AChE II substrate in the Y398 model with forces between 100 to 800 KJ·mol^−1^·nm^−1^ (Figure 5D), a constant force of 400 KJ·mol^−1^·nm^−1^ was chosen. Under this constant pulling force for a time period of up to 1 ns, this substrate could reach a site closer to S266 (0.4 nm) in the Y398 or Y398F models than in the Y398W or Y398G models where the distances converged to 0.5 and 0.6 nm, respectively (Figure 5E). We then explored the results of the conformational analysis between this Carbofuran-3-hydroxy substrate and four enzyme variants after applying a pulling force of 400 KJ·mol^−1^·nm^−1^ during 1 ns (Figure 9). On account of π–π stacking interactions between the substrate and Y/F398 or F399, the Carbofuran-3-hydroxy was oriented in a position that facilitated hydrogen bond formation with S266 and conducive to the occurrence of the carbamylation reaction (Figure 9). For the Y398G model, the absence of the side chain in the Y398G mutation, leads to rearrangements so that F399 replaces the structural function of Y398. However, the rotation of F399 is more restrained, which introduces steric hindrances so that the Carbofuran-3-hydroxy substrate remains at a larger distance from S266. For Y398W, the larger steric hindrance imposed by the larger side chain combined with the reduction of electronegativity of the Y398W mutation, seem to present the Carbofuran-3-hydroxy substrate in an unfavorable orientation for immediate catalysis by S266 (Figure 9).

**Figure 10:**
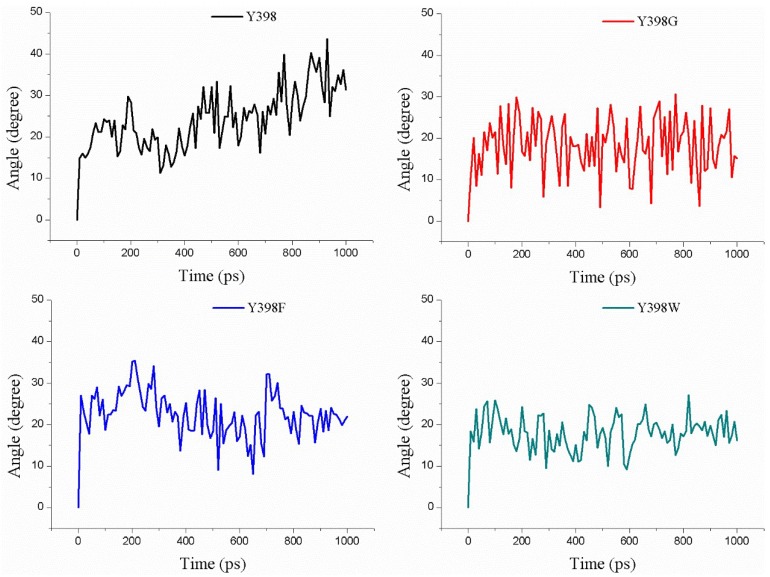
Rotation angle changes of Y/G/F/W398 within Carbofuran-3-hydroxy pulling process.

In Figure 10 we show the change in the rotation angle of Carbofuran-3-hydroxy during the pulling process through the different enzyme variants over a time period of 1 ns. Clearly, the Y398G and Y398W mutants remain less flexible compared to Y398 and Y398F. In addition, the calculation of the minimum size of the bottleneck residues is in agreement with the Y398 and Y398F keeping a “closed” conformation, which may present the substrate in a more unique and appropriate orientation for cleavage (Figure 7B). Overall, it appears that a proper size and a larger hydrophobicity of the bottleneck may increase the sensitivity of r*Bm*AChE II to pesticides.

## 3. Discussion

We employed the molecular modeled 3D-structure of r*Bm*AChE II to evolve the pesticide sensitivity of this enzyme. The experimental verification of the enzymatic activity of the mutants confirmed minor changes on sensitivity. The main reason for these mild changes on enzyme sensitivity might find its origin in the structural complexity of the enzyme mechanism. The r*Bm*AChE II is a macromolecule consisting of 604 amino acids. The active site of r*Bm*AChE II appears as a 20 Å deep narrow gorge with the catalytic triad at the bottom of the gorge and the edges being covered with aromatic residues, which provide a hydrophobic surface and negative charges [26]. They also comprise an acyl pocket and anionic site, with the peripheral anionic binding site (PAS) at the entrance of the gorge [27]. Half-way the gorge, the narrowest part referred to as bottleneck, is formed by amino acids F330 and Y121. The side chain of F330 seems to be flexible, whereas the position of Y121 is rather fixed, which might be important for controlling the entry of substrates to the catalytic triad [28].

Although AChE has been the subject of multiple crystal structure analyses, the crystal structure of r*Bm*AChE II is not available. In absence of crystal structure data of r*Bm*AChE II, the homology modeling of its structure is the best possible alternative. Furthermore, the exact AChE-ligand interactions and mechanism of catalysis remain elusive. By including multiple aspects and long timescales of molecular dynamic simulations in our study in conjunction with the generation of carefully selected point mutations and experimental verification of their enzymatic effects on substrate cleavage, we gained substantial insights into the enzyme mechanism. These studies required cutting edge equipment, software, and lengthy procedures [29]. Meanwhile, the umbrella sampling of steered molecular dynamic (SMD) was not performed in our research, which reduced the accuracy of SMD simulation. This is due to the limitation of our infrastructure and should be overcome in the further studies.

From prior studies of the AChE 3D structure, it appeared that many aromatic residues were decorating the wall of the bottleneck of the gorge. The study of Dougherty et al. [30] showed that the electron-rich π systems of aromatic residues could interact with the positive charge of the quaternary ammonium group of ACh. During the diffusion of the cationic ATChI, some aromatic residues indeed made important contributions. It was suggested that the aromatic residues might accelerate the cationic substrate by forming π-cation interactions [11]. As part of the aromatic guidance of the substrate, the side chain of Y398 constitutes the narrowest region of the active-site gorge. We therefore, decided to generate Y398G, Y398W, and Y398F variants of r*Bm*AChE II and to assess their effect on ATChI and other substrates. Remarkably, in comparison to the original r*Bm*AChE II enzyme, the Y398W mutant exhibited a higher activity towards ATChI. The conformational analysis indicated that the larger steric hindrance of the W aromatic side chain at the bottleneck together with the presence of larger number of hydrogen bonds with the ligand might encourage the fixation of ATChI in an appropriate orientation for enzymatic catalysis. Moreover, the large side chain of W pushes ATChI in close proximity of the oxyanion hole, forming hydrogen bonds with G179 and G180, which may further restrain the substrate flexibility. Previous research suggested that electrostatic steering might fasten the diffusion of positive charged ligands to the active site [31].

The situation for the Y398F mutant is quite different. Here the hydroxyl group of the side chain is eliminated, presenting a lower charge in this region, which might cause the significant reduced enzyme activity. In addition, the aromatic side chain at the bottleneck region is essential since Y398G exhibited a reduced sensitivity. Botti et al. [32] explained that the absence of the aromatic side chain at the bottleneck of the gorge would ruin the benefit induced by π–cation interaction.

Several studies tried to identify the residues of AChE that are responsible for the resistance to pesticides. Aiki et al. [33] investigated the F439W mutation in ACh E from *Tetranychus kanzawai* and concluded that F439, which is part of an acyl pocket and also of the bottleneck, might be critical in pesticide insensitivity. Likewise, the W331F mutation in *Branchiostoma floridae* ChE1 improved the sensitivity to pesticides, presuming that the width of the bottleneck had a significant role in pesticide inhibition [34]. These results are in accordance with our results. The enlargement of the bottleneck (Y398F) of r*Bm*AChE II will make the enzyme more suitable for binding bulky substrates. It was generally assumed that the bottleneck would lead to an open or closed state of the gorge, and a preferred closed state would control the substrate entry and exit by rotations of flexible side-chains [35,36]. Our research revealed that a more closed state of the bottleneck (as opposed with an open state for Y398G) with a well-defined unique rotamer might contribute substantially to the best possible presentation of the pesticide substrate to the catalytic triad. Indeed, keeping the bottleneck in a permanent “open” state of the bottleneck as with Y398G was contra productive for an efficient recognition of the substrate.

The effect of the Y398W mutation within r*Bm*AChE II on the carbofuran-3-hydroxy substrate seems to be less favorable due to an improper orientation of the substrate to interact with S266 (Figure 9). Similarly, according to Niu et al. [37], studying the binding of E2020 to TcAChE, a bulky substrate would adjust its conformation while crossing the bottleneck. Thus, we assume that the larger side chain (Y398W) within the bottleneck will alter the orientation of the Carbofuran-3-hydroxy for the same reason. This would explain the IC_50_ results where the Y398W mutant had a lower sensitivity to three pesticides but not to carbaryl. One study indicated that carbaryl comprising aromatic rings tended to adapt easier to mutations in the bottleneck, since it was not charged [38].

In this research, the mutation of Y398 could only affect the enzymatic activity of r*Bm*AChE II to a small extent. Since the catalytic triad of the enzyme is located at the bottom of a deep gorge within a large protein, the overall sensitivity of AChE to pesticides will be a synergistic effect of several amino acids. Likewise, the effect of the combined G228S and F439W mutations on AChE from *Tetranychus urticae* was demonstrated to possess a better effect on the insensitivity coefficient than the independent mutations [39].

In conclusion, we propose that for the positively charged and small substrate ATChI, a larger side chain at position 398 of r*Bm*AChE II improves the fixation of the substrate in an appropriate conformation for catalysis. For bulky substrates such as pesticides, the diffusion in the active site gorge may be related to the enlargement of the bottleneck by having proper orientations more easily. In addition, a more hydrophobic side chain at the bottleneck seemed to be beneficial for ligand diffusion.

## 4. Materials and Methods

*E. coli* Top10 and *Pichia pastoris* GS115 were used as host cells for cloning and recombinant protein expression. The TIANamp Yeast DNA Kit was obtained from Tiangen Biotech (Beijing) Co., LTD., (Beijing, China), and Mut Express II Fast Mutagenesis Kit V2 was obtained from Vazyme Biotech Co., LTD (Nanjing, China). DNA polymerase and restriction enzymes were purchased from Thermo Fisher Scientific (Shanghai, China). Primers were synthesis by Ruibiotech Co., LTD., (Beijing, China). Pesticide standards were obtained from Beijing Tanmo Quality Inspection Technology Co., LTD (www.gbw-china.com). ATChI was provided by Tokyo Chemical Industry Co., Ltd. The 5,5′-Dithiobis (2-nitrobenzoic acid) (DTNB) substrate was from Guangzhou Jianyang Biotechnology Co., Ltd., (Guangzhou, China).

### 4.1. 3D-Structure Homology Modeling and Docking

The r*Bm*AChE II amino acid sequence was uploaded to the online server Swiss-model (https://swissmodel.expasy.org/). Using *Drosophila Melanogaster* AChE crystal structure as template, the server returned three possible structural models, ranked 1 to 3, for the r*Bm*AChE II. Then the online server PROCHECK (http://services.mbi.ucla.edu/PROCHECK/) was employed to examine the quality of the models. Subsequently, docking of the three models with substrate ATChI, OPs and CBs pesticides (including phoxim, malathion, parathion, methamidophos, chlorpyritos, methomyl, aldicarb, carbaryl, carbofuran, and carbofuran-3-hydroxy) was performed with the LeadIT software. Based on the correlation coefficient of the docking scores and IC_50_ as standards, one docking within the three models was chosen as the most appropriate.

**Figure 11:**
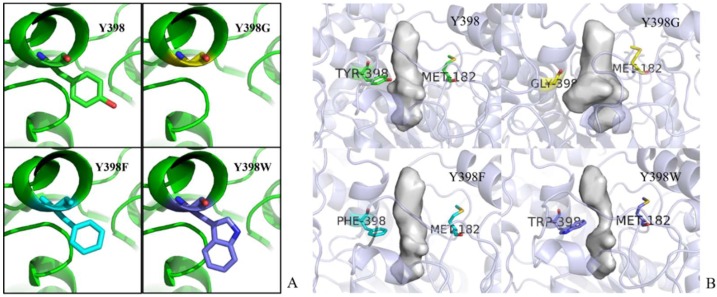
(**A**) Y398 mutants. The r*Bm*AChE II backbone is represented as a green ribbon; Y398, Y398G, Y398F, Y398W side chain carbons are shown by sticks in green, yellow, cyan, and magenta, respectively; red stick in Y398 refers to the phenolic hydroxyl group, dark blue in Y398W refers to the secondary amine; and (**B**) Pocket size. Bottleneck residues were presented in sticks, and pocket size is shown in contour surfaces.

The interactions within the dockings were analyzed, and Y398 was noted to form a hydrogen bond between ATChI and all pesticides. Therefore, Y398 was targeted for substitution, in independent reactions, by three different amino acids (Y398G, Y398F, and Y398W). The Y398F substitution provides the same benzene ring without the phenolic hydroxyl group (similar steric hindrance but no hydrogen bond), Y398W will expose a larger hydrophobic surface and a polar N atom (more steric hindrance and able to form a hydrogen bond), and the Y398F substitution removes the side-chain (less steric hindrance and no hydrogen bond) (Figure 11A). We conducted the homology modeling on the three mutants with the Swiss-model server. The No. 1 model of each mutant was chosen for further investigations. The catalytic pocket of the mutants was analyzed by the online server POCASA 1.1 (http://altair.sci.hokudai.ac.jp/g6/index-e.html).

### 4.2. Mutated rBmAChE II Plasmid Construction and Transformation

Using the vector pPIC9K- r*Bm*AChE II (constructed previously in our lab with the predicted N-terminal signal peptide removed) as template, three pairs of primer were designed containing the Y398 mutations (Table 2). PCR mutation, *DpnI* digestion and recombinant reaction were according to the manufacturers of the Mut Express II Fast Mutagenesis Kit V2 (Vazyme Biotech Co., LTD (Nanjing, China)). Recombinant vectors were transformed into *E. coli* Top10 cells and sequenced.

The constructed pPIC9K-*Bm*ace2, the wild type and its mutants, were linearized with *SalI* and electrotransformed into *Pichia p.* GS115. The transformants were numbered and expressed separately in 96-deep-well plates. The enzyme activity of each clone was measured.

### 4.3. Screening of Bmace Copy Number by q-PCR

Clones showing enzyme activities were chosen and the *Bm*ace copy number was quantified by q-PCR relative to the internal reference GAPDH gene (primer sequences shown in Table 1). Standard curves with two pairs of primers were made as follows: genomic DNA of r*Bm*AChE II expressing GS115 clones was extracted using TIANamp Yeast DNA Kit, adjusted to 10 ng/μL and serially diluted (10^−1^, 10^−2^, 10^−3^, 10^−4^, and 10^−5^) before being employed as q-PCR template. Templates (1 μL) of each dilution were added to the reaction mix consisting of 12.5 μL SYBR *Premix Ex Taq II* (Takara, Beijing, China), 1 μL of both forward and reverse primer and 9.5 μL ddH_2_O. Q-PCR was performed in triplicate using the CFX96 Real-Time PCR Detection System Bio-rad, Guangzhou, China) and following settings: 95 °C for 30 s; 40 cycles of amplification at 95 °C for 5 s, 55 °C for 30 s; and 95 °C for 10 s, followed by the Melt Curve steps between 65 °C and 95 °C, at 0.5 °C/cycle.

Genome DNA extracts of the chosen clones and the q-PCR reaction mixtures were as above. The Q-PCR program was 95 °C for 30 s; 40 cycles of amplification at 95 °C for 5 s, 55 °C for 30 s, 72 °C for 30 s, and 72 °C for 10 min. Each sample was mixed with two pairs of primers (bmace-qPCR and GAPDH-qPCR pairs, Table 1) and repeated three times. The relative copy number was calculated based on 2^−ΔΔ*C*t^ method. Clones of the three mutants and wild type that had a similar relative copy number were chosen for further studies. 

### 4.4. Protein Expression

After q-PCR screening, four types of *Pichia p.* GS115 with different plasmids were cultured in BMGY (1% yeast extract, 2% peptone, 100 mM phosphate buffer pH 6.0, 1.34% YNB, 1% glycerol, and 0.1% ampicillin) and expressed in BMMY (1% yeast extract, 2% peptone, 100 mM phosphate buffer pH 7.0, 1.34% YNB, 1% methanol, and 0.1% ampicillin). The *Pichia p.* GS115 without plasmid was used as control. Briefly, strains were cultured on MD plating medium, containing 1.34% yeast nitrogen base (YNB) and 2% glucose. Single colonies were grown at 30 °C, 250 rpm for 12 h in 10 mL yeast extract peptone dextrose (YPD) medium (1% yeast extract, 2% peptone) supplemented with 2% glucose and 0.1% ampicillin. Cells were 1:100 diluted in BMGY and incubated at 30 °C and shaken at 250 rpm. After 24 h, cells were harvested by centrifugation at 4 °C, 4000 rpm for 5 min. The cell pellet was resuspended in BMMY, to reach an OD_600_ of around 45 (high density expression). Expression was performed at 28 °C, shaken at 250 rpm for 4 days and supplemented with 1% methanol and 0.1% ampicillin, every day. Production of 4 days expression was centrifuged at 8000 rpm for 5 min at 4 °C and the supernatant was collected as crude enzyme. Each type of *Pichia p.* GS115 was performed in triplicate.

### 4.5. Enzyme Activity and Inhibition by Physostigmine

The r*Bm*AChE II enzyme activity was evaluated according to the Ellman method [40]. Each well of a 96-wells microtiter plate contained 150 μL of a 50 mM phosphate buffer, 90 μL crude enzyme and 30 μL 7.5 mM chromogenic reagent DTNB. The volume of enzyme was substituted with an equal volume of phosphate buffer in a well for a blank control. The mixtures were incubated at 37 °C for 10 min followed by adding 10 mM substrate ATChI. The changes of OD_405_ at 37 °C were recorded immediately after adding the substrate. The amount of enzyme capable of catalyzing 1 nmol ATChI in 1 minute was defined as 1 unit, and was calculated as follows:

Enzyme activity (U/mL) = (ΔA405 × *V* × *a*)/(ε × *d* × *T* × *V*e) × 10^6^

(ΔA405: change in 405 nm absorbance; *V*: volume of the reaction mixture; *a*: dilution ratio of enzyme; ε: extinction coefficient, 1.36 × 10^4^ L/(mol•cm); d: optical path; *T*: response time; *V*e: enzyme volume).

The remaining activity was also tested after inhibition with physostigmine. 30 mL 0.1 mg/mL physostigmine was added to the mixture (120 μL, 50 mM phosphate buffer, 90 μL crude enzyme, and 30 μL, 7.5 mM DTNB) and incubated at 37 °C for 10 min. The remaining activity was detected as above.

### 4.6. Analysis of Pesticide Sensitivity

To compare the sensitivity of the mutated enzymes towards pesticides, wells were prepared containing 60 μL 50 mM phosphate buffer, 90 μL pesticides at different concentration, 90 μL crude enzyme and 30 μL 7.5 mM DTNB. Enzyme activity in the presence of pesticides was calculated as above. Standard curves were fitted and the IC_50_ was measured.

### 4.7. Molecular Dynamic Simulation

Molecular dynamic (MD) simulation, including MD and steered molecular dynamic simulation (SMD), was conducted with GROMACS software. More precisely, r*Bm*AChE II model 1 protein was solvated in a cubic box at 0.5 nm of the protein, and neutralized with 20 Na^+^ ions. Energy minimizing (EM) of the whole system was run with energy convergence, followed by respectively 500 ps position restraints (PR), canonical ensemble (NVT) and constant pressure isothermal ensemble (NPT). The system then was equilibrated with 10 ns MD. The Y398G, Y398F, and Y398W mutant models were conducted similarly. Ligand (ATChI, Carbofuran-3-hydroxy) was added at the entry site of the active site cleft of different receptors, having an orientation consistent with LeadIT docking results.

After a pre-equilibration of the complexes as above, SMD was performed. We set the catalytic site S266 (S200 in Tc) and the ligand (ATChI or Carbofuran-3-hydroxy) as two pulling groups and approaching these molecules with a constant force (*K*) from 100–800 KJ·mol^−1^·nm^−1^. A proper pulling force was chosen for further studies. Under the constant force (K) of 200 and 400 KJ·mol^−1^·nm^−1^, ATChI and Carbofuran-3-hydroxy had a proper entering status.

The Y398G, Y398F, and Y398W were also pre-equilibrated under the same conditions. Complexes including model_1-ATChI, model_1-Carbofuran-3-hydroxy, Y398W-ATChI, Y398W-Carbofuran-3-hydroxy, Y398F-ATChI, and Y398G-Carbofuran-3-hydroxy were run with the most appropriate pulling force. The change in distances as a function of time was recorded.

We analyzed the conformations of the SMD results through VMD (Visual molecular dynamics) software and Pymol. Within the pulling process, the distance changes and the minimum distance of the two target residues within the bottleneck (Y/G/F/W398 and M182), the rotation angle of Y/G/F/W398 and the amount of hydrogen bonds between protein and ligand, were calculated by GROMACS analytical procedures.

## Figures and Tables

**Table 1:** q-PCR results of clones with similar relative copy number.

No.	GAP *C*t	*Bm*ace *C*t	2^−ΔΔ*C*t^
Y-33	19.46	19.46	1.002
G-15	19.60	19.70	0.933
F-16	18.63	18.67	0.975
W-10	19.09	19.18	0.939

Y, G, F, and W refers to the residue at position 398. Numbers behind the residue type is the clone number.

**Table 2:** Pair of primers to mutagenize codon 398 and primers for quantitative PCR of *Bm*ace and GAPDH.

Gene	Primer
*Y398G*	5′-GGGTACCggtTTCTTGTTGTACGACTTCTTGGACTAC-3′ 5′-ACAAGAAaccGGTACCCTCGTCTTGGTTAGAACC-3′
*Y398F*	5′-GGGTACCttcTTCTTGTTGTACGACTTCTTGGACTAC-3′ 5′-ACAAGAAgaaGGTACCCTCGTCTTGGTTAGAACC-3′
*Y398W*	5′-GGGTACCtggTTCTTGTTGTACGACTTCTTGGACTAC-3′ 5′-ACAAGAAccaGGTACCCTCGTCTTGGTTAGAACC-3′
*bmace*-qPCR	5′-GCTATCAAGAACGCTACGAAT-3′
*GAPDH*-qPCR	5′-AACTCTGTATTGCATAGAAGC-3′ 5′-CGTCGGTATTAACGGTTTCG-3′ 5′-GCTTGTAAGCCTTGTGGGT-3′

Note: the lowercase letters correspond to the mutated codon.

## Abbreviations

AChE	acetylcholinesterase
r*Bm*AChE II	recombinant *Bombyx mori* type II acetylcholinesterase
OP	organophosphorus
CB	Carbamate
*P. pastoris*	*Pichia pastoris*
BMGY	buffered glycerol-complex medium
BMMY	buffered methanol-complex medium
MD	minimal dextrose medium
YPD	yeast peptone dextrose
bmace	Bombyx mori acetylcholinesterase gene
DTNB	5,5′-dithiobis-(2-nitrobenzoic acid)
ATChI	acetylthiocholine iodide
COM	center of mass
MD	molecular dynamic
SMD	steered molecular dynamic
